# Impact of the New Malaysian Cigarette Pack Warnings on Smokers’ Awareness of Health Risks and Interest in Quitting Smoking

**DOI:** 10.3390/ijerph7114089

**Published:** 2010-11-22

**Authors:** Ahmed I. Fathelrahman, Maizurah Omar, Rahmat Awang, K. Michael Cummings, Ron Borland, Ahmad Shalihin Bin Mohd Samin

**Affiliations:** 1 The Clearing House for Tobacco Control, the National Poison Center, Universiti Sains Malaysia, Minden 11800 Pulau Pinang, Malaysia; E-Mails: maizurahomar@yahoo.com (M.O.); rahmatawang@yahoo.com (R.A.); asms@notes.usm.my (A.S.B.M.S.); 2 Ministry of Health-Khartoum State, Khartoum, Post Office Box 1517, Sudan; 3 Department of Health Behavior, Roswell Park Cancer Institute, Elm and Carlton Streets, Buffalo, NY 14263, USA; E-Mail: Michael.Cummings@RoswellPark.org; 4 Cancer Control Research Institute, the Cancer Council Victoria, 1 Rathdowne St, Carlton, Victoria 3053, Australia; E-Mail: ron.borland@cancervic.org.au

**Keywords:** pictorial cigarette package warning labels, smoking behavior, awareness of health risk, interest in quitting, Malaysia, adult smokers, randomized trial, questionnaire, knowledge score

## Abstract

The objective of this research was to compare the response of adult smokers in Malaysia to newly proposed pictorial cigarette warnings against the current text-only warnings. The study population included 140 adult male smokers who were enrolled in a randomized trial to view either the new pictorial warnings (intervention) or the old text-only warnings (control). Participants completed pre-exposure and post-exposure questionnaires that assessed their awareness of the health risks of smoking, response to the package warnings, and interest in quitting smoking. Exposure to the pictorial warnings resulted in increased awareness of the risks of smoking, stronger behavioral response to the warnings and increased interest in quitting smoking. The new warnings in Malaysia will increase smokers’ knowledge of the adverse health effects of smoking and have a positive effect on interest in quitting.

## 1. Introduction

In 2000, Canada became the first country to mandate large graphic pictorial warnings on cigarette packs. Each design was composed of; a general warning theme, text-based information, a picture, and self-efficacy oriented health messages [1,2]. Since Canada first adopted its graphic pack warnings, many other countries have done the same [2–7]. The number of countries adopting graphic warnings is increasing (see http://www.tobaccolabels.ca/healthwarnings for the latest number).

Several published studies, mostly conducted in high income countries, have confirmed the superiority of graphic pack warnings compared to text-based warnings in informing the public about the risks of smoking and stimulating interesting in quitting smoking [6,8–12]. Risk perception has been found to be affected by socioeconomic factors, among which are education and income [13]. This highlights the importance of studying the effect of cigarette warning labels in middle- and low-income countries to ascertain either universality or diversity of the impact of such warnings across countries with different levels of access to knowledge. A study in Mexico, a middle income country, found that smokers respond to warnings in a similar way to smokers in high income countries [14]. In the same context, Fathelrahman and associates [15] showed that Malaysian smokers responded to textural warnings in a fashion comparable to that identified in the West, so we might expect a high degree of generalization. However, it is important to test this hypothesis and also very useful to see if reactions to isolated exposures mirror those to repeated exposures as happens once warnings are introduced.

**Figure 1:**
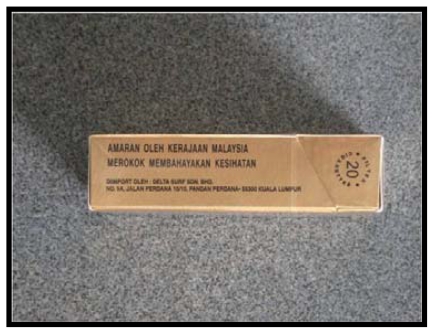
The text-only cigarette package warning label used in Malaysia before the implementation of the new labeling policy starting from January 2009.

Figure 1 shows the standard pack warning on cigarettes sold in Malaysia before January 2009. The single text-only message stated: “warning by the Malaysian government; smoking is hazardous to health”. Malaysia ratified the World Health Organization’s Framework Convention on Tobacco Control in September 2005, which obligated them to adopt an enhanced tobacco product warning system [16]. Malaysia, like many other countries, adopted a rotating series of graphic pictorial warnings labels beginning in January 2009.

This paper presents results from an evaluation undertaken study just prior to the appearance of the new graphic warnings on cigarette packs to gauge how the new warnings might impact awareness of the health risks of smoking, response to the package warnings, and interest in quitting smoking among a sample of Malaysian adult current smokers.

## 2. Methodology

### 2.1. Design of Study

A two-group randomized design was used to compare the impact of the new graphic warning labels against the original text based pack warnings. Participants were 140 male Malaysian adults (18 years and older), at least weekly smokers, who completed the study (from 174 who commenced). Subjects were recruited as volunteers from different places within Penang State to the Western North of Malaysia during the period from the May to December 2008. Allocation to study group was initially by coin toss, then alternating group.

### 2.2. Procedures

All subjects enrolled in the study were asked to participate in two sessions. During the first visit, participants were told that “the study is evaluating cigarette pack warning labels and that they (*i.e.*, participant) are going to answer certain questions related to smoking and that they will be asked to return to see certain materials before being asked again to answer a second questionnaire”. Following consent, they were asked to complete a questionnaire which asked about their smoking history, demographic characteristics, questions about awareness of the health risks of smoking, exposure and response to health warnings on cigarettes, and interest in stopping smoking. It was written in Malay with an English translation made available if preferred.

**Figure 2:**
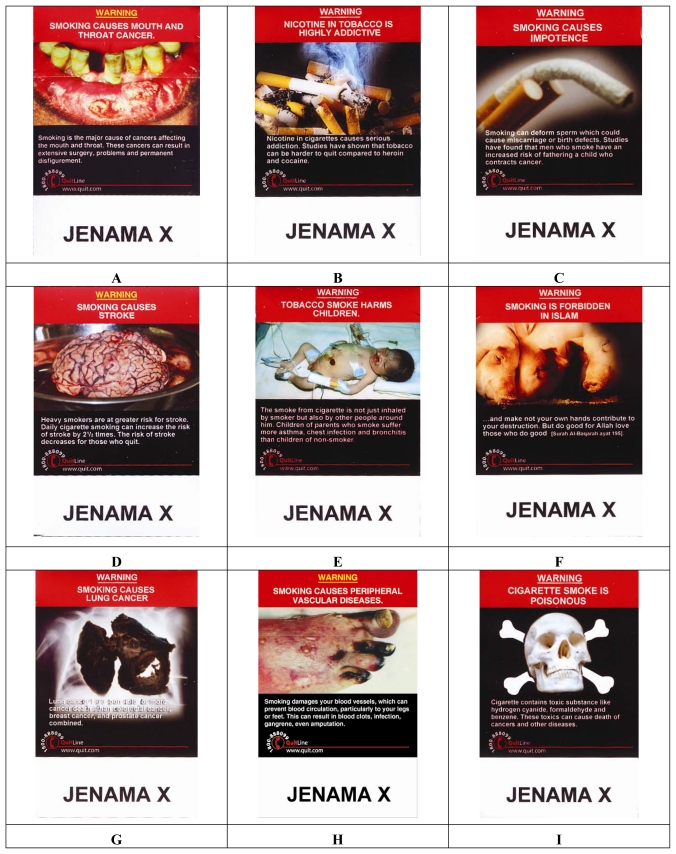
The nine designs used as mock-ups in the study.

In the second session, approximately one week later, participants were provided with either mocked-ups cigarette packs showing the new graphic health warnings as illustrated in Figure 2, or a set of local cigarette brands using the standard text based warning, as shown in Figure 1. The graphic packs were prepared to resemble the new designs of the cigarette pack warning labels proposed by Malaysian government. The text-based information printed besides the depicting pictures in the graphic packs was written in both languages Malay (front side of the pack) and English (back side). In both groups, participants were given the packs all at the same time and instructed to take a few minutes to examine them. After about five minutes the packs were collected from respondents and they completed a second questionnaire reassessing their awareness of the health risks of smoking, response to health warnings on cigarettes, and interest in stopping smoking. The entire session took less than 30 minutes.

Of the 174 respondents enrolled in the study at baseline, 140 completed the second survey. Only those participants who completed both the baseline and follow-up surveys are included in analyses presented here. As shown in Table 1, the comparison of the smoking characteristics of the subjects assessed at baseline revealed no significant differences between those assigned to either group. In addition, there were no differences with regards to the age, gender, ethnic group, religion, higher level of education, annual household income and employment status.

The intent of separating the two sessions was to allow a brief washout time between completing the baseline and follow-up surveys, which asked many of the same questions. Study participants were reimbursed for transportation costs to and from the study location. In addition subjects who completed both sessions were given a free study t-shirt (cost about RM 30) as an incentive. The study was approved by the USM ethical committee (FWA00007718, IRB00004494).

### 2.3. Outcome Measures

The three main outcomes were: (1) pre-/post-changes in awareness of the health risks of smoking; (2) pre-/post- changes in responses to warning labels; and (3) pre-/post-changes in interest in stopping smoking. Each is described below.

#### 2.3.1. Awareness of health risks

Participants were asked which of 13 different health conditions were related to smoking. Health conditions included; stroke in smokers (blood clots in the brains), impotence in male smokers, lung cancer in smokers, decay in the lungs of smokers, stained teeth in smokers, peripheral vascular diseases, diabetes, bronchial asthma in children of smokers, addiction to smokers, poisoning with toxic substances, a lot of serious diseases in smokers and in people around them who do not smoke, mouth cancer in smokers and gangrene in the legs or feet of smokers. If the person answered “no” to diabetes they were scored as having a correct answer. A total awareness score was computed by assigning a “1” to all correct responses (range 0–13).

#### 2.3.2. Response to warning labels

Participants were asked five questions about how they respond to cigarette product warnings labels. The five questions were: (1) to what extent, if at all, do you read or look closely at labels (not at all, a little, somewhat and a lot)? (2) To what extent, if at all, do those health warnings make you think about the health risks (not at all, a little, somewhat and a lot)? (3) To what extent, if at all, do the health warnings on the cigarette pack designs make you more likely to quit smoking (not at all, a little, somewhat and a lot)? (4) To what extent, if at all do the warning labels cause you to stop from having a cigarette (never, once, a few times and many times); and (5) to what extent if at all, do you avoid looking at the warning labels (yes and no/unsure)? Questions 2 and 3 were combined into a composite variable similar to Borland *et al.* [11] with three levels (2 = a lot for both; 1 = a lot for one; and 0 = all other combinations). At baseline session, the five questions were about how the smokers respond to the warning labels commonly available in the market at time of study (*i.e.*, text-only warning). In the second session, the questions were directed towards the warning labels viewed in the study (pictorial warnings for the intervention and text-only warnings for the control).

#### 2.3.3. Interest in quitting

Participants were asked if they are planning to quit smoking in the future with four possible answers; within the next month, within the next six months, sometime in the future beyond six months or not planning to quit.

#### 2.3.4. Statistical analyses

Both descriptive and comparative analyses were performed. For comparative analyses, differences between experimental and control groups were tested by independent student t-test for the scale data and by Chi-square or McNemar statistics for categorical data when comparing pre-post measures within groups. Multiple logistic and multiple linear regressions were used to predict the effect of the warning labels on three outcomes; post-treatment knowledge, post-treatment cognitive responses and post-treatment interest in quitting. P < 0.05 was considered statistically significant.

## 3. Results

### 3.1. Awareness of Health Risks

Exposure to the pictorial health warnings was associated with a significant increase in awareness of the health risks of smoking (see Table 2) Before viewing the pack warnings, knowledge scores did not differ significantly between the two groups, with any difference favouring the controls. After viewing the warnings, average awareness score increased 1.81 points (1.05 to 2.58) among those exposed to the pictorial warnings compared to a slight decline (−0.21 [−0.87 to 0.44]) among the controls.

This included significant increases among the intervention group in correct responses to “smoking cause peripheral vascular diseases” (p = 0.035 ), “smoking cause bronchial asthma in children of smokers” (p = 0.036), “smoking cause addiction to smokers” (p = 0.012), “smoking cause poisoning with toxic substances” (p = 0.001), “smoking cause a lot of serious diseases in smokers and in people around them who do not smoke” (p = 0.003), “smoking cause mouth cancer in smokers” (p = 0.002), and “smoking cause gangrene in the legs or feet of smokers” (p < 0.001).

### 3.2. Response to Warning Labels

Table 3 summarizes the changes in the cognitive and behavioral responses to pack labels. Exposure to the pictorial pack warnings was associated with significant increases over the control text warning in smokers both thinking about the harm of smoking (Think-harm), and about quitting smoking (Think-quit), in avoiding looking at or thinking about the pack warnings, and forgoing have a cigarette because of the warning, but no change in likelihood of reading or looking closely at them.

### 3.3. Interest in Quitting

As shown in Table 3, interest in quitting increased significantly more in those exposed to the pictorial warnings.

### 3.4. Showing the Effect of the Pictorial Warning Labels (Intervention) Using Regression Analyses

Table 4 shows that the pictorial warning labels (intervention) was associated with greater increases in the three outcomes, post-treatment knowledge, post-treatment cognitive responses and post-treatment interest in quitting. The regression analysis for each outcome controlled for the demographic variables age, race, addiction score, and baseline measures of knowledge, cognitive responses, quit interest and self-efficacy.

## 4. Discussion

This study demonstrates that the new Malaysian health warnings will have beneficial effects on knowledge and concerns about the harms of smoking and interest in quitting, at least in the short term. These findings are consistent with previous studies showing the benefits of large pictorial cigarette pack warnings over text-based warnings in high income countries [11,12,17] and another middleincome country [14]. The main strength of this study is that it was a randomized trial, thus demonstrating a causal relationship between exposure to the warnings and the changes in knowledge and smoking-related concerns. The main limitations are that we only assessed immediate reactions, and only assessed the pictorial warnings as a group. This means we cannot say anything about longer term effects, or on the specific effects of particular warnings, except for relevant knowledge increases. We also note that, in some cases, we used questions that covered a longer period, but it is clear from the mode of responding that participants took it to mean what they expected they would do, rather than what they did do. The sample used here was also a convenience sample, not representative of smokers in Malaysia. Nevertheless, there was nothing special about the sample to suggest that their reactions would systematically differ to reactions of other Malaysian smokers.

The finding of knowledge increases across most of the health effects is consistent with respondents taking in the information that was provided. Those with no significant increases were ones with very high baseline levels, and the small increases were not significant. One item, on asthma, was not a headline health effect, but was mentioned in the supplementary text to the headline warning “warning: tobacco smoke harms children”, suggesting that respondents read at least some of the detailed information.

When considered in conjunction with the findings of population-based studies of representative samples from other countries which show long term predictive effects, we can conclude that this research strengthens the argument that these population-wide effects are causal, and that the same kinds of long term beneficial effects of the new warnings found elsewhere are likely to occur in Malaysia. Graphic health warnings both can improve public knowledge, and work to encourage cessation through the generation of concern stimulated by the emotionally charged messages. The value of graphic warnings is that they make the potential of smoking to cause disease more real to the smoker, and thus are better at stimulating them to think about acting to avoid this risk. In this regard, it is important to point out that the increase in reported avoidance of warnings found here is a positive, not a negative, predictor of subsequent quitting [12].

Analog studies of the kind used here can be used to predict likely impact of warnings when implemented, given that they mirror population-level effects of prolonged exposure. Coupled with the existing evidence, this study demonstrates that the new Malaysian warnings are likely to lead to better knowledge of health effects of smoking [17], and will lead to more of the reactions to warnings that have been prospectively shown to predict making quit attempts [11,12].

## Figures and Tables

**Table 1:** Smoking characteristics of adult smokers who participated in the study.

Item	Frequency (%)		P value
	Control (N = 70)	Intervention (N = 70)	
Number of cigarettes smoked per day a			0.798 *
(0) ≤10 cigarettes	30 (42.9)	25 (36.2)	
(1) 11–20	34 (48.6)	38 (55.1)	
(2) 21–30	4 (5.7)	3 (4.3)	
(3) >30	2 (2.9)	3 (4.3)	
First cigarette how soon after waking b			0.361*
(0) After 60 minutes	32 (45.7)	27 (39.7)	
(1) 30 to 60 minutes	18 (25.7)	13 (19.1)	
(2) 6 to 30 minutes	11 (15.7)	12 (17.6)	
(3) Within 5 minutes	9 (12.9)	16 (23.5)	
Do you smoke more frequently during the first hours after waking c			0.115*
(0) No	55 (78.6)	46 (66.7)	
(1) Yes	15 (21.4)	23 (33.3)	
Which cigarette would you hate most to give up d			0.176*
(0) All	41 (58.6)	32 (47.1)	
(1) First one in the morning	29 (41.4)	36 (52.9)	
Do you smoke if you are so ill e			0.532*
(0) No	57 (81.4)	54 (77.1)	
(1) Yes	13 (18.6)	16 (22.9)	
Do you find it difficult to refrain from smoking f			0.957*
(0) No	50 (71.4)	49 (71.0)	
(1) Yes	20 (28.6)	20 (29.0)	
Average score (mean ± SD) on nicotine dependence a	2.74 ± 2.14	3.46 ± 2.47	0.070**
Nicotine dependence g□			0.410*
Low nicotine dependence	46 (65.7)	38 (56.7)	
Medium nicotine dependence	15 (21.4)	15 (22.4)	
High nicotine dependence	9 (12.9)	14 (20.9)	
Perceived addiction (how addicted to cigarette) h			0.692*
Not at all addicted	16 (22.9)	19 (27.1)	
Somewhat addicted	42 (60.0)	40 (57.1)	
Very addicted	5 (7.1)	7 (10.0)	
Can’t say	7 (10.0)	4 (5.7)	
About how many years have you been smoking i			0.316*
<1 year	6 (8.6)	2 (2.9)	
1–5 years	9 (12.9)	14 (20.6)	
6–10 years	20 (28.6)	22 (32.4)	
>10 years	35 (50.0)	30 (44.1)	
Have you ever tried to quit smoking l			0.334*
No	8 (11.4)	12 (17.1)	
Yes	62 (88.6)	58 (82.9)	
In the last month, have you stubbed out a cigarette before you finished it because you thought about the harm of smoking m			0.158*
No	37 (52.9)	44 (64.7)	
Yes	33 (47.1)	24 (35.3)	

(%): column percentage

* Chi-square

** independent T-test;

□ according to Fagerstrom questionnaire, 0–3 (low nicotine dependence), 4–5 (medium nicotine dependence), 6–10 (high nicotine dependence);

a N = 139 smokers,

b N = 138 smokers,

c N = 139 smokers,

d N = 138 smokers,

e N = 140 smokers,

f N = 139 smokers;

g N =137 smokers;

h N =140 smokers,

i N = 138 smokers,

l N = 140 smokers,

m N = 138 smokers.

**Table 2:** Differences in the scores on knowledge of health risks between the adult smokers in the intervention and the control groups at baseline and after treatment and the change in those scores.

Scores on perceptions	Mean score (*95% CI*)		Mean (*95% CI*)	P-value
	Control (N = 70)	Intervention (N = 70)	Difference*	
Knowledge at baseline (total scores)	8.04 (7.28–8.80)	7.47 (6.75–8.19)	−0.57 (−1.61–0.47)	0.278
Knowledge after intervention (total scores)	7.83 (7.09–8.57)	9.28 (8.63–9.94)	1.46 (0.48–2.44)	0.004
The change in knowledge scores	−0.21 (−0.87–0.44)	1.81 (1.05–2.58)	2.03 (1.03–3.03)	<0.001

**Table 3:** Summary of the changes in the responses toward the warning labels from baseline measurement to after-treatment among the adult smokers in the intervention and the control groups.

Smokers’ responses	Control (N = 70)			Intervention (N = 70)		
	Before Frequency (%)	After Frequency (%)	P Value *	Before Frequency (%)	After Frequency (%)	P value*
Reading or looking closely at labels a lot	13 (18.6)	4 (5.7)	0.035	9 (13.0)	12 (17.4)	0.607
Think-harm	8 (11.4)	8 (11.4)	>0.999	8 (11.6)	20 (29.0)	0.004
Think-quit	14 (20.0)	17 (24.3)	0.664	15 (21.7)	27 (39.1)	0.017
Foregoing smoking a cigarette	22 (31.4)	22 (31.4)	>0.999	17 (24.6)	40 (58.0)	<0.001
Avoid looking at or thinking about the label	14 (20.0)	29 (41.4)	0.003	6 (8.7)	34 (49.3)	<0.001
Quit intention						
No interest	22 (31.4)	20 (28.6)	0.242	25 (35.7)	12 (17.1)	0.003
Interested beyond 6 months	29 (41.4)	30 (42.9)		30 (42.9)	28 (40.0)	
Interested within 6 months	7 (10.0)	10 (14.3)		6 (8.6)	17 (24.3)	
Interested within the next month	12 (17.1)	10 (14.3)		9 (12.9)	13 (18.6)	

Percentages were total (*i.e.*, from the number of subjects in the intervention or the control); frequencies reported for binary variables were the positive responses only.

* McNemar test.

**Table 4:** The effect of the exposure to the pictorial warning labels (intervention) on post-treatment knowledge, post-treatment cognitive responses and post-treatment interest in quitting.

Covariates	Post-treatment knowledge B *(95% CI )*^a^	Post-treatment cognitive responses B *(95% CI)*^a^	Post-treatment interest in quitting OR *(95% CI)*^b^
Age (scale)	−0.03 (−0.07–0.01) NS	0.01 (−0.01–0.02) NS	1.04 (0.99–1.08) NS
Race (categorical)	0.15 (−1.01–1.31) NS	0.11 (−0.20–0.41) NS	NS
Score on nicotine addiction (scale)	−0.18 (−0.38–0.02) NS	−0.04 (−0.10–0.01) NS	0.80 (0.64–1.00) NS
Baseline knowledge score (scale)	0.46 (0.30–0.61) ***	−0.02 (−0.06–0.012) NS	1.07 (0.90–1.26) NS
Baseline score on cognitive responses (scale)	−0.12 (−1.06–0.81) NS	0.38 (0.13–0.63) **	1.06 (0.33–3.37) NS
Baseline quit interest (categorical)	0.60 (−0.44–1.64) NS	0.12 (−0.15–0.39) NS	7.91 (2.47–25.34)**
Baseline self-efficacy (categorical)	0.25 (−0.99–1.48) NS	0.12 (−0.21–0.45) NS	0.60 (0.13–2.73) NS
Exposure to pictorial warning (*i.e.*, control versus intervention)	1.92 (1.03–2.81) ***	0.33 (0.10–0.57) **	2.79 (1.04–7.48)*

a Multiple linear regression;

b multiple logistic regression;

NS statistically not significant;

* P < 0.05,

** P < 0.01;

*** P < 0.001; all categorical variables are binary except race which includes Malay, Chinese, Indian & Others.
